# Effect of an education and activation programme on functional limitations and patient-perceived recovery in acute and sub-acute shoulder complaints – a randomised clinical trial

**DOI:** 10.1186/1471-2474-8-112

**Published:** 2007-11-15

**Authors:** Camiel De Bruijn, Rob de Bie, Jacques Geraets, Marielle Goossens, Wim van den Heuvel, Geert van der Heijden, Math Candel, Geert-Jan Dinant

**Affiliations:** 1 Institute for Rehabilitation Research, PO box 192, 6430 AD, Hoensbroek, The Netherlands; 2 Maastricht University, Department of General Practice and Care and Public Health Research Institute, PO box 616, 6200 MD, Maastricht, The Netherlands; 3 Maastricht University, Department of Epidemiology, PO box 616, 6200 MD, The Netherlands; 4 Maastricht University, Department of Medical, Clinical and Experimental Psychology, PO box 616, 6200 MD, The Netherlands; 5 University Medical Centre, Julius Center for Health Sciences and Primary Care, PO box 85500, 3508 GA, Utrecht, The Netherlands; 6 Maastricht University, Department of Statistics and Methodology, PO box 616, 6200 MD, The Netherlands

## Abstract

**Background:**

The education and activation programme (EAP) aims at coping with psychosocial determinants to prevent the development of chronic shoulder complaints (SCs). The effect of the EAP on functional limitations and patient-perceived recovery after 6 and 26 weeks is evaluated in a randomised clinical trial.

**Methods:**

Patients with SCs present at rest or elicited by movement and lasting no longer than 3 months were allocated at random to either EAP as an addition to usual care (UC), or to UC only. Measurements were taken at baseline and after 6 and 26 weeks and were analysed by means of multilevel analysis for the group effect. EAP was administered by GPs or by an ambulant therapist (CDB). Patients in the UC group were given UC by their own GP.

**Results:**

Multilevel analysis failed to show a significant effect of the EAP on either functional limitations or patient-perceived recovery. Analysis showed coincidentally a relation between catastrophising at baseline and functional limitations.

**Conclusion:**

The EAP has no significant effect on the outcome of SCs after 6 and 26 weeks. The relation between catastrophising at baseline and functional limitations suggests that an intervention focusing specifically on catastrophising may be more successful in reducing functional limitations in the long term. Further research is however needed to evaluate the effect of catastrophising at baseline on the course of SCs.

**Trial registration:**

Current Controlled Trials ISRCTN71777817

## Background

Psychological and social factors are known to play a role in the development and persistence of chronic musculoskeletal diseases [1-3]. Furthermore, previous studies showed that therapies aimed at coping with psychosocial determinants are promising interventions to prevent musculoskeletal pain from becoming chronic [4-8]. Up till now, usual care (UC) in patients with shoulder complaints (SCs) in the Netherlands has mainly focused on the biomedical determinants mentioned in the clinical guidelines of the Dutch College of General Practitioners [9]. Since half of all newly presented episodes of SCs in general practice last for at least six months, a therapy aimed at coping with psychosocial determinants may reduce the proportion of SCs that become chronic [10].

We have therefore developed an education and activation programme (EAP) aimed at coping with psychosocial determinants to prevent the development of chronic SCs. Psychosocial determinants influence cognitions and behaviours. The EAP attempts to steer these cognitions and behaviours in the desired direction to avoid the development of inadequate cognitions and maladaptive behaviours. In this context, cognitions refer to the way patients think about their SCs and what these complaints mean to them, in terms of thoughts, beliefs, attitudes and self-efficacy expectations [11], whereas behaviour refers to the patients' observable actions [12].

Our hypothesis is that in the acute and sub-acute stages of the SCs, cognitions and behaviours are easily susceptible to modification, which means that the EAP can be administered in a brief intervention by specially trained general practitioners (GPs) or a trained ambulant therapist. A randomised clinical trial was set up to evaluate the effect of the EAP as an addition to UC, compared to UC alone, on patient-perceived recovery and changes in functional limitations of activities of daily living after 6 weeks and 26 weeks [13]. This paper presents the results of the randomised clinical trial as regards functional limitations and patient-perceived recovery after 6 and 26 weeks. The trial is part of a national study on shoulder complaints in general practice, which includes a prognostic cohort study with three randomised clinical trials in subcohorts. This study is funded by The Netherlands Organization for Health Research and Development (grant number 940-31-085).

## Methods

### Study design

In our randomised clinical trial, patients were allocated at random to either EAP as an addition to UC, or to UC only. Measurements were taken at baseline and after 6 and 26 weeks by a self administered questionnaire. The 6 and 26 weeks questionnaires were sent and returned by mail. The 6 weeks measurement provided information on the immediate effect of the EAP as the EAP had to be administered within the six week period after the baseline measurement. The 26 weeks measurement provided information on the long term effect of the EAP. EAP was administered by GPs or by an ambulant therapist (CDB) if no EAP-trained GP was available near a patient's home. All GPs who provided EAP attended a three-hour training session, in which the EAP was introduced and role-plays were used to train the proper administration of EAP. The GPs received a training manual during this session in which the principles of the EAP were summarized. Patients in the UC group were given UC by their own GP, unless their GP had attended the EAP training. In that case, UC was administered by a colleague from the same GP group practice, to avoid contamination. The design of this study has been described in detail in a previous publication [13]. The Medical Ethics Committee of the Institute for Rehabilitations Research in association with Rehabilitation Foundation Limburg has approved this randomised clinical trial.

### Patients and procedure

Eligible patients had consulted their own GP or responded to advertisements in local newspapers calling on people with a new and untreated episode of SCs that had lasted less than three months and produced complaints at rest or complaints elicited by shoulder movement. Patients were included if they were older than 18 years and living in the south of the Netherlands. Additional inclusion and exclusion criteria are given in table 1.

**Table 1:** Inclusion and exclusion criteria

**Inclusion criteria**	**Exclusion criteria**
• SC present at rest or elicited by movement	• Prior fractures and/or surgery of the shoulder
• SC episode lasting no longer than 3 months	• (Suspected) referred pain from internal organs
• First episode of SC for 12 months	• SCs with a confirmed extrinsic cause
• Newly presented episode (no prior consultations or treatments for this episode in the previous three months)	• Inability to complete a questionnaire independently
• Older than 18 years	• Presence of dementia or other severe psychiatric abnormalities
• Living in the south of the Netherlands	

The patient recruitment procedure in the consultation room was designed to minimize the time needed by the GP, since lack of time is often mentioned as one of the main barriers when recruiting patients in general practice [14]. GPs pointed out the existence of the EAP trial to patients with newly presented SCs, checked the inclusion and exclusion criteria and forwarded the patients' personal data by fax to the research centre. The patients had to give written permission for their personal data to be forwarded to the research centre. The GPs were advised to refer patients to the research centre for further information about the EAP trial. Patients responding to the advertisements were first screened by telephone for inclusion and exclusion criteria. Patients meeting the inclusion criteria were subsequently visited by an independent GP involved in the EAP trial (EAP-GP). This EAP-GP provided the patients with a regular consultation for SCs. Personal data of eligible patients were forwarded to the research centre. In both recruitment strategies, the research assistant contacted patients within 2 weeks and took care of final inclusion and randomisation. The baseline questionnaire was handed out and after completion returned to the research assistant.

Blocked and concealed randomisation with blocks of 4 patients was used to allocate patients to either the EAP group or the UC group. An independent researcher used a computer-generated random sequence table to randomise the patients in each block. The seals of the prepared, numbered, opaque, sealed envelopes containing the treatment group were broken by the research assistant after eligibility had been verified and the patient had given written informed consent.

### Blinding

Neither the patients nor the GPs, nor the trained ambulant therapist, could be blinded for the allocated treatment. The ambulant therapist was also the researcher coordinating the randomised clinical trial and conducting the data analysis, but he was blinded for treatment allocation during the data analysis. The allocation code was kept by an independent researcher (JG) and was revealed only after data analysis had been completed.

### Interventions

The focus of EAP is to maintain or induce the proper cognitions by education and to stimulate adequate behaviour by means of advice on activities of daily living, using principles of operant conditioning (11). The programme consists of an educational and an activation part.

The educational part of the EAP consists of tailored information intended to take away the worries and answer questions patient may have regarding their SCs. Special care is taken to structure the information and advice that patients receive from other individuals in their social and health care environment. The information and advice are tailored to the patients' thoughts. Preconceptions on SCs are identified and altered if they are incorrect or reinforced if they are correct. The final aim of the educational part of the EAP is to provide patients with a realistic idea of their prognosis and the effect of treatment.

The activation part aims to assist patients in the continuation or resumption of activities affected by the SCs, despite the pain. The adverse effects of inactivity are discussed with the patients and activities that patients indicate to be affected by the SCs are closely monitored during the subsequent consultations. Schedules are set for the resumption or gradual increase of these activities, using a time-contingent approach, which means that the resumption or increase of activities occurs irrespective of pain experience but according to preset goals in time.

The EAP consists of a minimum of two sessions and a maximum of six follow-up sessions over a period of six weeks. Each session can last up to 20 minutes.

UC was administered according to the clinical guidelines of the Dutch College of General Practitioners [9].

### Outcome Measures

The first primary outcome measure, assessed at 6 and 26 weeks after randomisation, was patient-perceived recovery (PPR) [10]. Patients were considered recovered when they reported to be 'much improved' or 'fully recovered', on a 7-point ordinal scale, six weeks after randomisation.

The second primary outcome measure was a change in functional limitations of activities of daily living. This variable was assessed by the 16-item shoulder disability questionnaire (SDQ)[15], with a standardised scoring range of 0 to 100. A lower score on this questionnaire implies lower levels of functional limitations.

Several psychosocial variables were assessed at baseline. Anxiety, depression, somatisation and distress were assessed using the four-dimensional complaint list [16]. Catastrophising and coping were assessed by 6-level subscales of the Pain Coping and Cognition List (1: completely disagree; 6: completely agree) [17]. Mean subscale scores of 1 were classified as 'very low' (code = 0), scores between 2 and 6 were classified as elevated (code = 1) [17]. Other specific disease variables recorded at baseline were pain intensity, measured on a 10-point visual analogue scale; onset (quick or gradual); affected shoulder and having had prior episodes of SCs lasting at least 1 week.

### Sample size

About half of all newly presented episodes of SC in general practice are reported to last for at least six months. A number needed to treat of 4.5 after 26 weeks is considered clinically relevant. This implies an absolute reduction of 22% of the proportion of patients with SC after 26 weeks. With a two-sided alpha of 0.05 and a statistical power (1-β) of 0.80, 82 patients per treatment group were needed to detect a difference in favour of EAP compared to UC after 26 weeks.

### Data analysis

The baseline variables of the treatment groups were compared using chi-square tests and an independent samples t-test. Significant differences in baseline variables were considered to be potential confounders. The statistical data analysis was carried out according to the 'intention-to-treat' principle.

Patients attending the same GP cannot be assumed to be fully independent. Similarly, different observations for the same patient with SCs cannot be assumed to be independent either. Multilevel analysis was used to address this dependency due to clustering of data. The effect of treatment group (EAP group = 1; UC group = 0) was analyzed by means of linear multilevel analysis if SDQ was the outcome variable and logistic multilevel analysis if patient-perceived recovery was the outcome variable. Three levels of variance were distinguished: GPs, subjects and measurements.

In the linear multilevel analysis, baseline SDQ scores were entered into the model to adjust for differences at baseline. Since none of the patients were recovered at baseline, no adjustment was needed for patient-perceived recovery at baseline in the logistic multilevel analysis. Time of measuring was represented in the model by two dummy variables for the measurements at 6 and 26 weeks. Potential confounders were also entered into the linear and logistic model as independent variables. Finally, the interaction effect between the treatment group and the time of measuring was also included in the model.

The multilevel analyses resulted in estimates (and standard errors) of the fixed and random effects. Likelihood ratio (LR) test statistics were used to determine whether the estimates were statistically significant (p < 0.05) in the linear multilevel analyses. Wald chi-square tests were used to determine the statistical significance of the estimates in the logistic multilevel analyses. Estimates that did not reach the required level of significance were excluded from the model in a top-down procedure, except for the intervention variable, leaving out the least significant estimates first. For the logistic multilevel analysis, these estimates were converted to odds ratios with their 95% confidence intervals. All multilevel analyses were performed with MLwiN (version 1.10) [18,19].

## Results

### Patients

**Figure 1:**
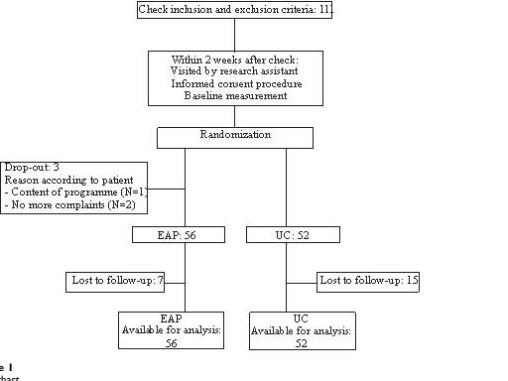
Flow chart.

GPs referred a total of 133 patients for participation in the EAP trial, 74 of whom were actually included in the trial. In total, 64 patients responded to the advertisements. Of these, 44 met the selection criteria, as ascertained by a telephone interview. The subsequent visit by the EAP-GP resulted in the final inclusion of 37 patients. A total of 111 patients were thus eventually recruited to participate in the study (figure 1). Three patients dropped out after randomisation without receiving any treatment, and were therefore excluded from further analysis. After 6 weeks, complete data was available for 40 patients (77%) in the UC group and 48 patients (86%) in the EAP group. After 26 weeks, complete data was available for 35 patients (67%) in the UC group and 44 (79%) patients in the EAP group.

### Baseline variables

Table 2 shows that there were no statistically significant differences between the two treatment groups at baseline for any of the variables except catastrophising. The effect of baseline differences in catastrophising was evaluated by entering this variable into the multilevel analysis models.

**Table 2:** Baseline variables

	**UC**	**EAP**	**p-value**
Number	52	56	
**Demographic variables**			
Age (years) (SD)	49.9 (11.7)	48.4 (16.2)	0.572
Gender ♂ (%)	44	36	0.366
**Specific disease variables**			
Pain intensity T0 (mean + SD)	5.2 (2.3)	5.3 (2.2)	0.887
Onset (quick) (%)	44	54	0.286
Affected shoulder (Left/right/both) (%)	42-52-6	41-57-2	0.538
Prior episodes of SCs lasting at least 1 week (% yes)	42	49	0.482
**Outcome variable**			
SDQ score at baseline (mean and SD)	60.8 (24.1)	67.1 (24.0)	0.175
**Psychosocial variables**			
Catastrophising (% very low)	49	27	**0.022**
Coping (% very low)	12	8	0.463
Distress categories (low-medium-high; %)	79-17-4	80-18-2	0.815
Depression categories (low-medium-high; %)	94-2-4	91-7-2	0.357
Anxiety categories (low-medium-high; %)	100-0-0	100-0-0	-
Somatisation (low-medium-high; %)	85-11-4	82-18-0	0.231

### Multilevel analyses

Estimates with their standard error and levels of significance are presented in table 3 for the linear multilevel analyses of the SDQ scores. The interaction terms between the two dummy variables and the group variable did not reach significance and were excluded from the final model in the top-down procedure. This implies that the potential effect of treatment group was similar at both post measurements. In the final model, treatment group turned out to have no effect at either of the measurements. Catastrophising at baseline and baseline SDQ scores were significantly and positively related to SDQ scores at both post measurements. Time dummy 1 and time dummy 2 were significantly and negatively related to SDQ scores, suggesting that SDQ score, representing the level of functional limitations, decreases as time progresses.

**Table 3:** Results of the linear multilevel analysis of SDQ

**Variable**	**Estimate (SE)**	**p-value**
**Fixed parameter**		
Treatment group	3.44(4.98)	0.4916
Time dummy 1 (6 weeks)^1^	-8.18(3.24)	0.0116^2^
Time dummy 2 (26 weeks)^3^	-17.26(4.03)	0.0000^2^
Catastrophising at baseline	6.84(5.10)	0.0000
SDQ score at baseline	0.53(0.11)	0.0000
**Random effects**		
**Variance at patient level**		
□ Time dummy1	819.03(133.67)	0.0000^2^
□ Time dummy 2	1227.20(205.03)	0.0000^2^

1 This variable has the code 1 for 6 weeks and 0 otherwise

2 Based on the Wald statistic

3 This variable has the code 1 for 26 weeks and 0 otherwise

Estimates with their standard errors and levels of significance for the logistic multilevel analyses of the PPR are presented in table 4. Data were interpreted by converting estimates to odds ratios (ORs). The top-down procedure resulted in the exclusion of the interaction terms from the logistic model. This implies that the potential effect of treatment group was similar for both post measurements. In the final analysis model, the treatment group had a non-significant (p = 0.1784) effect on patient-perceived recovery. A significant effect was found for time dummy 1 and time dummy 2, indicating a significant effect on PPR of the time elapsed since baseline. Baseline levels of catastrophising did not have a significant effect on the PPR and were excluded from the final analysis model.

**Table 4:** Results of the logistic multilevel analysis of patient-perceived recovery

**Variable**	**Estimate (SE)**	**OR**	**95% CI**	**p-value**
**Fixed parameter**				
Treatment group	0.85(0.63)	2.34	[0.68;8.00]	0.1784^1^
Time dummy 1 (6 weeks)^2^	1.24(0.45)	3.46	[1.43;8.33]	0.0056^1^
Time dummy 2 (26 weeks)^3^	2.60(0.49)	13.46	[5.16;35.16]	0.0000^1^

1 Based on the Wald statistic

2 This variable has the code 1 for 6 weeks and 0 otherwise

3 This variable has the code 1 for 26 weeks and 0 otherwise

## Discussion

Multilevel analysis using either Shoulder Disability Questionnaire (SDQ) scores or PPR as the outcome variable failed to show a significant effect of the EAP. Baseline levels of catastrophising were significantly and positively related to functional limitations. Patient-perceived recovery (PPR), on the other hand, was not significantly related to baseline levels of catastrophising. A significant effect of time was found for both outcome variables.

The positive and significant correlation between functional limitations and elevated levels of catastrophising at baseline suggests the existence of a relation between the two. Such a relation is plausible, as catastrophising refers to 'an exaggerated negative orientation toward pain stimuli and pain experience' [20-22]. This may cause patients with SCs to be more reluctant to use their shoulder, resulting in increased functional limitations.

In view of this, it is remarkable that no relation was found between catastrophising and patient-perceived recovery, since negative orientation is expected to affect the perception of the SCs as well. Such a relation between catastrophising and the chronic pain experience has indeed been found in patients with low back pain [23-27]. Furthermore, Kuijpers et al. also found an association between catastrophising at baseline and PPR after 6 weeks in patients with SCs. A possible explanation for the absence of a relation in our analysis may have been the dichotomous nature of the outcome measure making it difficult to detect a relation between catastrophising and patient-perceived recovery.

The relation between catastrophising and functional limitations at baseline and the absence of a relation with patient-perceived recovery raises some questions. Why does the EAP have no effect on functional limitations, even though one of its aims is to change catastrophising cognitions? On the other hand, the absence of a relation with patient-perceived recovery raises the question whether it is worthwhile to intervene on catastrophising cognitions if no relation is found between the two although it is also possible that the absence of such a relation can be attributed to the dichotomous nature of the outcome measure.

At the start of this study, little was known about the specific effect of psychosocial determinants in SCs, although interventions aiming to modify these determinants, such as cognitive behavioural programs were considered to be promising for musculoskeletal pain in general [4-8]. We developed the EAP to fill a gap in UC, which focuses mainly on biomedical determinants. The EAP uses techniques used in cognitive behavioural therapy that were expected to benefit patients in the early stages of SCs. Furthermore, GPs had to be able to apply these techniques after a brief training whereas cognitive behavioural programs are usually administered by specialized therapists.

The lack of information on specific psychosocial determinants of SCs made us develop a generic intervention addressing several psychosocial determinants, of which catastrophising cognitions is only one. This generic nature of the EAP may be the reason that we found no effect on outcome. Although this study shows that catastrophising at baseline is related to one of the outcome measures, namely functional limitations, catastrophising cognitions are not the main focus of the EAP. Another reason for the absence of an effect of the EAP may be that other, unknown, determinants are more closely related to SCs. This view is supported by the absence of a relation between catastrophising at baseline and patient-perceived recovery, suggesting that the relation between catastrophising and outcome is not as straightforward as expected.

It should be noted that the relation between catastrophising at baseline and functional limitations was a coincidental finding of this study. Baseline levels of catastrophising were initially entered into the multilevel models to adjust for baseline differences between study groups. A study by van der Windt et al. showed that higher levels of catastrophising in patients with longer symptom duration are significantly associated with persistent symptoms [28]. Further study is needed to evaluate the effect of catastrophising in SCs and the possibilities of interventions focusing on catastrophising.

The significant effect of time on outcome suggests that SCs are likely to improve over time regardless of the intervention. This positive effect of time is found in other studies as well [10,29-31]. Identifying patients with this favourable natural course in the early stages of SCs appears to be difficult [32]. Otherwise, the effectiveness of any intervention could be improved by focussing on the patients at risk. Further study is needed to identify factors predicting a favourable natural course in the early stages.

Analysis of videotaped consultations showed that not all key features of the EAP were applied by the trained GPs [33]. Furthermore, GPs administering UC were already including some key features exclusively attributed to the EAP in their UC. This may have reduced the contrast between the treatment groups.

Ours appears to be the first study addressing psychosocial determinants in patients with acute and sub-acute SCs in general practice. A recent study evaluating a similar intervention in patients with low back pain in general practice also found no effect [34].

Outcome measures were collected using self reported questionnaires. Both patient perceived recovery and functional limitations may thus be influenced by the state of mind of the patient reporting the outcome. From this point of view, outcome measures reflect the patient's subjective perspective whereas an objective measure would be unbiased by the patient's state of mind. We preferred a subjective outcome over an objective outcome as we wanted to evaluate the effectiveness of the intervention from the patient's perspective.

The effect of the EAP on total costs related to SCs is evaluated using bootstrap analysis [35]. This analysis showed that the EAP is more effective but at higher costs. Furuthermore, healthcare utilisation showed no difference between the study groups. It should be noted that a bootstrap analysis is specifically designed to evaluate the cost-effectiveness of an intervention. The multilevel analysis presented in this paper is able to determine the isolated effect of the EAP.

Recruitment by GPs fell short of our expectations. Therefore we opted for an alternative strategy using advertisements. This introduces the risk of selection bias. However, a comparison of baseline values (not presented) showed no differences between the recruitment methods. Even more, entering recruitment method as a variable in the multilevel analysis showed no significant effect of recruitment method on outcome (not presented in this paper). It appeared that GPs recruiting patients were less accurate in checking the inclusion and exclusion criteria. This resulted in a higher exclusion rate of patients referred for participation in the trial by GPs.

It may be questioned whether a brief training is sufficient to enable GPs to administer the EAP as prescribed. Our hypothesis was that patients' cognitions and behaviours in the acute and sub-acute stages of the SCs are susceptible to modification. We may have underestimated, however, that cognitions and behaviours by the GPs towards SCs were less susceptible to modification, and that the GPs might require a more intense training to become thoroughly acquainted with the key features of the EAP. This may have affected the quality of the EAP administered by the GPs.

Based on our sample size calculation, we needed 82 patients per treatment group. However, patient recruitment fell short of our expectations. Eventually, 56 patients were included in the EAP group and 52 patients were included in the UC group. This resulted in a reduction of the power of this study. A post-hoc power calculation indicated a statistical power of 0.63, compared to the intended power of 0.80. This power reduction increases the risk of a 'false negative' finding in this study.

Although complete data was available for 67% of the patients in the UC group and 79% of the patients in the EAP group, multilevel analysis made it possible to use all data available of the 52 patients in the UC group and 56 patients in the EAP group. The difference in available complete data between groups may be influenced by the fact that patients were not blinded. It is likely that patients in the UC group were less inclined to complete participation in the study after being allocated to UC.

## Conclusion

Multilevel analysis shows that the EAP has no significant effect on the outcome of SCs. A coincidental finding of this study was the relation between catastrophising at baseline and functional limitations. This relation suggests that an intervention focusing specifically on catastrophising may be more successful in reducing functional limitations in the long term. In contrast, the EAP addresses catastrophising as one of several psychosocial determinants of SCs. The effect of an intervention focusing on catastrophising may be improved by selecting patients with elevated levels of catastrophising at baseline. Further research is needed to evaluate the effect of catastrophising at baseline on the course of SCs.

## Competing interests

The author(s) declare that they have no competing interests.

## Authors' contributions

CDB drafted the manuscript, participated in the design of the study and performed the statistical analysis. RDB and GJD participated in the coordination of the study and helped to draft the manuscript. JG and MG participated in the coordination of the study and the design of the questionnaires. WVDH critically reviewed the methods used in this study and helped to draft the manuscript. GVDH conceived of the study and participated in the design of the study and reviewed the manuscript. MC participated in the statistical analysis. All authors read and approved the final manuscript.

## Pre-publication history

The pre-publication history for this paper can be accessed here:


